# Supplementary material for: Deletion of Thioredoxin-Interacting Protein (TXNIP) Abrogates High Fat Diet-Induced Retinal Leukostasis, Barrier Dysfunction and Microvascular Degeneration in a Mouse Obesity Model
Source: Int J Mol Sci. 2020 Jun 1;21(11):3983. doi: 10.3390/ijms21113983 (PMC7312035; doi:10.3390/ijms21113983)
Supplement: Supplementary file 1 [file ijms-21-03983-s001.pdf]

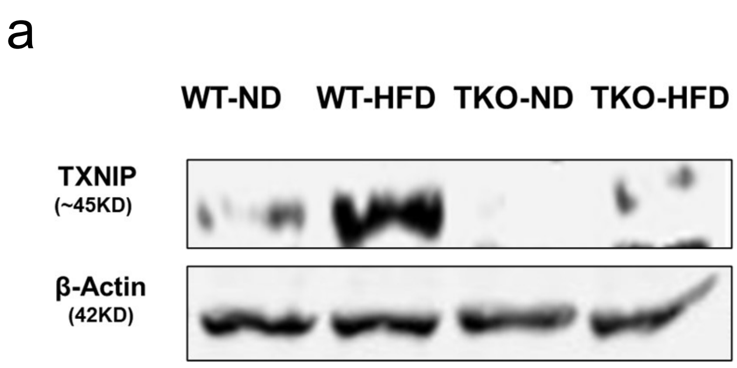

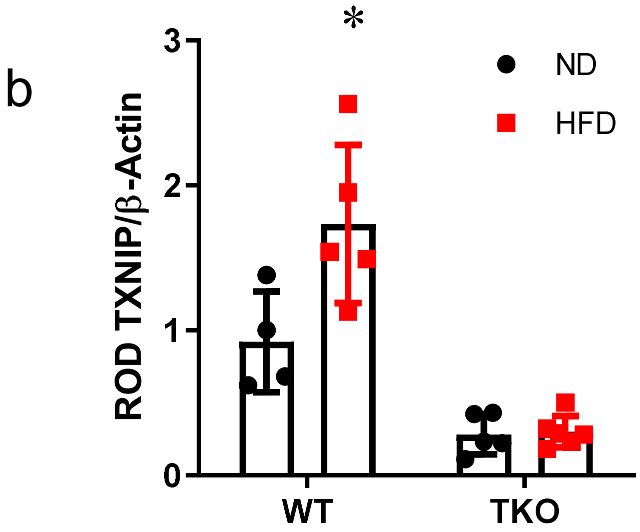

Supplementary-Figure-1. High fat diet (HFD) triggers retinal TXNIP expression after 8weeks. (a) Representative blots of TXNIP and β-Actin and (b) statistical analysis for retinal TXNIP expression showing a 1.7-fold increase in TXNIP expression in WT when compared to normal diet (ND) controls, (n=4-5; * P-value <0.05 vs all groups). TXNIP knockout mice showed minimal expression of TXNIP. Two–way ANOVA analysis showed significant interaction between genotype (TXNIP deletion VS WT) and between type of diet (HFD vs ND).

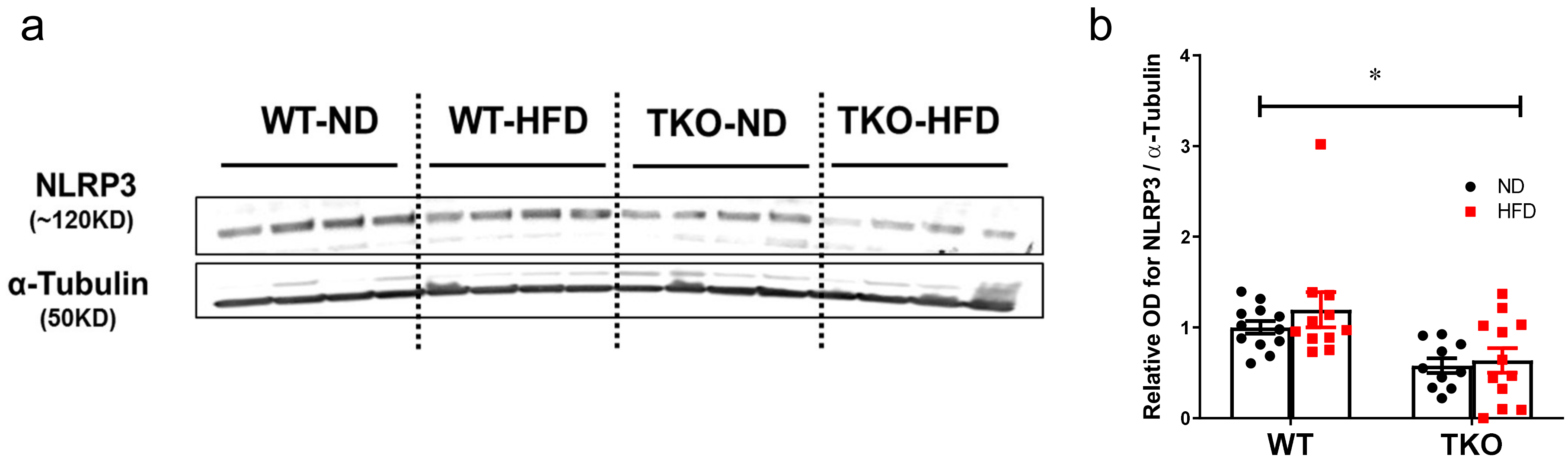

Supplementary-Figure-2. Representative WB blots (a) and statistical analyses of protein expression of retinal NLRP3 (b) showed a trend towards higher levels of NLRP3 in WT-HFD group compared with WT-ND. In contrast, TKO-ND and TKO-HFD groups showed lower levels of all target protein expression compared with WT-ND group, respectively. Two-way ANOVA showed significant interaction between the genotype (TKO vs WT) across NLRP3 expression (n=12 mice/group; *P< 0.05 vs other groups).

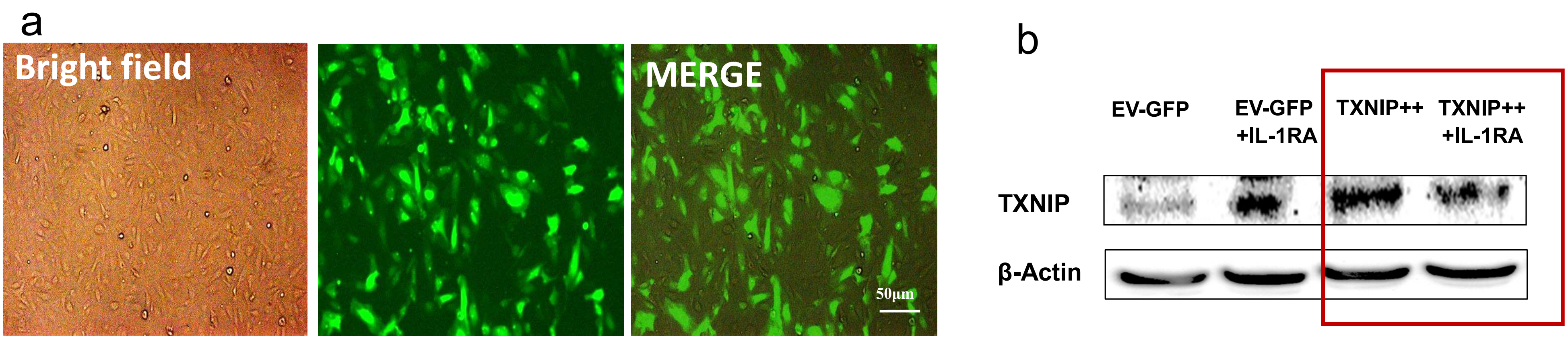

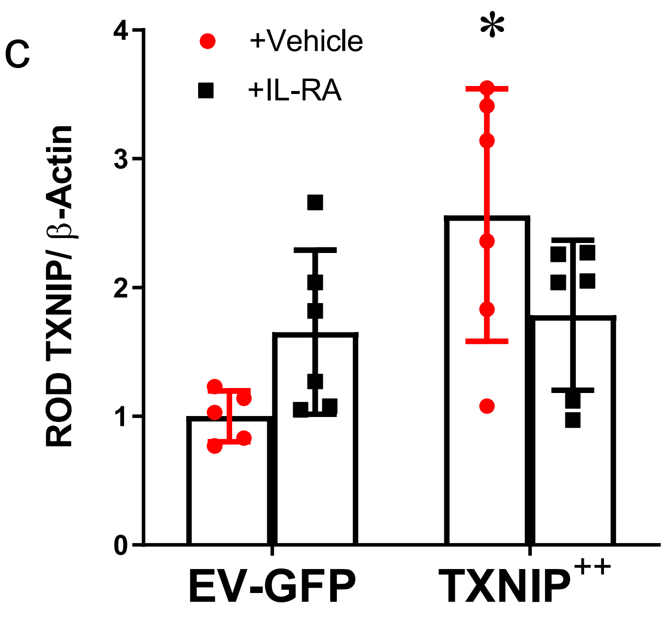

Supplementary-Figure-3. Representative pictures for the transfection efficiency of TXNIP plasmid labeled with green fluorescence protein (GFP) or empty vector (EV-GFP) in human retinal endothelial cells (REC). (a) Representative images for bright field, and green fluorescence and merge showing a transfection efficiency of 80-90% as indicated by the ratio of GFP expressing cells to the total number of REC. (b) Representative blots of TXNIP and β-Actin and & (c) statistical analysis for TXNIP overexpression in human REC cultures ($TXNIP^{++}$) showing a 2.5-fold increase in TXNIP expression when compared to empty vector (EV-GFP) controls, (n=5-6; * P-value <0.05 vs EV-GFP).

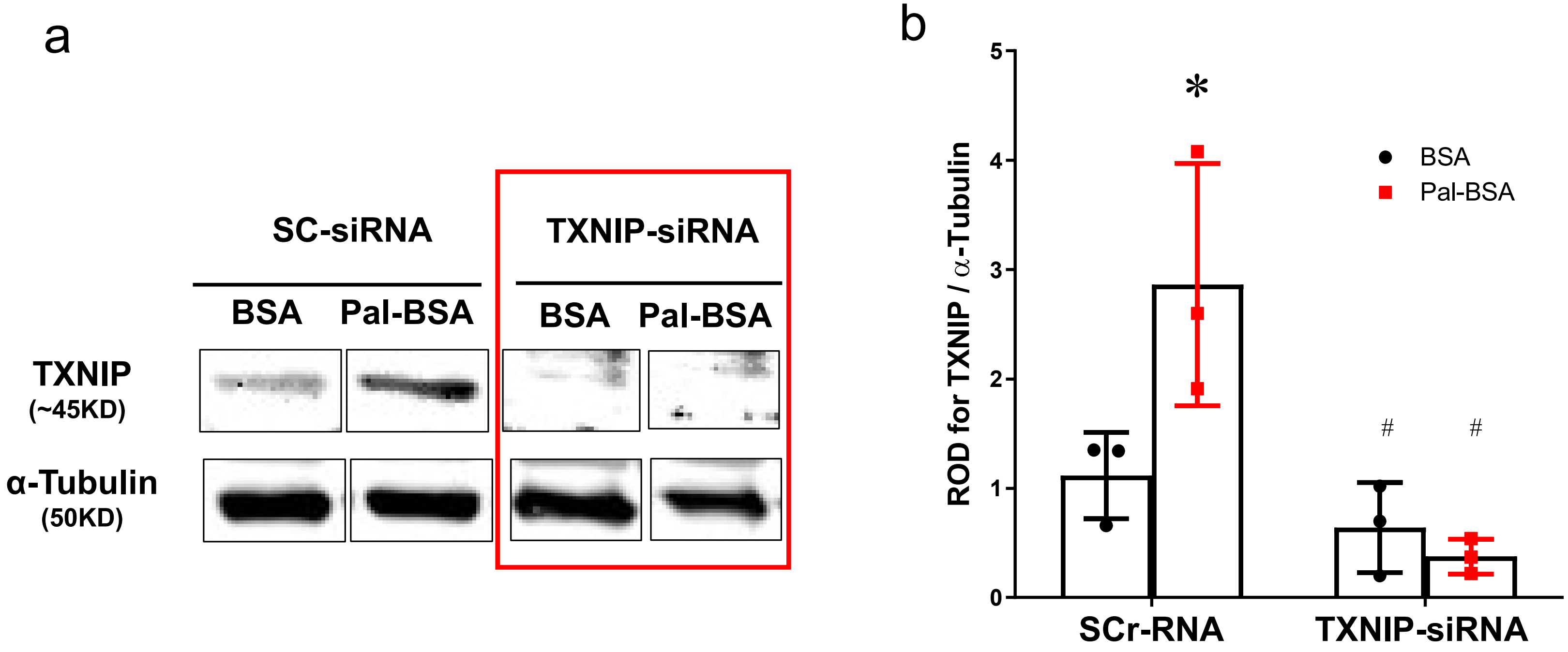

Supplementary-Figure-4. (a) Representative blots of TXNIP and α-tubulin and (b) statistical analysis for silencing TXNIP expression using siRNA in human REC cultures showing a 3-fold increase in TXNIP expression in response to palmitate (Pal-BSA) when compared to BSA-control in the cells transduced with scrambled RNA. Transduction with siRNA against TXNIP significantly abrogated TXNIP expression when compared to cells transduced with scrambled RNA (n=3; * P-value <0.05 vs other groups, # vs Scr-RNA).
